# Outcome of MRSA carriers in neurological early rehabilitation

**DOI:** 10.1186/1471-2377-14-34

**Published:** 2014-02-21

**Authors:** Jens D Rollnik

**Affiliations:** 1 Institute for Neurorehabilitational Research (InFo), BDH-Clinic Hessisch Oldendorf, Teaching Hospital of Hannover Medical School, BDH-Clinic Hessisch Oldendorf, Greitstr. 18-28, 31840 Hess, Oldendorf, Germany

## Abstract

**Background:**

Colonization with MRSA is believed to have deteriorating effects on neurological rehabilitation patients because MRSA carriers need to be isolated.

**Methods:**

Medical records of neurological early rehabilitation patients (most of them after stroke) admitted to a large rehabilitation facility in Northern Germany in 2010 have been carefully reviewed with respect to MRSA status, outcome variables (functional independence), morbidity, and length of stay (LOS).

**Results:**

74/569 (13.0%) patients were MRSA positive on admission. MRSA carriers had a significantly longer LOS in early neurological rehabilitation (63.7 (37.1) vs. 25.8 (24.5) days, p < 0.001), worse functional status on admission (Barthel index (BI) 13.6 (9.9) vs. 25.6 (24.1), p < 0.001), worse Glasgow Coma Scale (9.5 (3.2) vs. 12.0 (3.3), p < 0.001), more co-diagnoses (20.5 (5.1) vs. 13.3 (5.5), p < 0.001), and higher Patient Clinical Complexity Levels (PCCL). The outcome was significantly worse among MRSA positive patients (BI 25.5 (21.2) vs. 47.4 (31.0), p < 0.001; Early Rehabilitation Index −47.3 (51.4) vs. -26.0 (35.4), p < 0.001). Isolated patients had slightly less therapy per day (131.6 (16.6) vs. 140.2 (18.7) min/day, p < 0.001), but the overall sum of therapy was significantly larger in the MRSA positive group due to longer LOS.

**Conclusions:**

Functional recovery of MRSA carriers in early neurological rehabilitation is worse than in MRSA negative patients. Poorer outcome is not resulting from isolation (less therapy) but from functional status and higher morbidity on admission.

## Background

Several studies suggest that colonization with methicillin resistant Staphylococcus aureus (MRSA) is a growing problem in rehabilitation and nursing facilities. In 1982, the rate of MRSA colonized patients in a U. S. rehabilitation center was only 0.44% [1]. Eight years later, 20.6% of veterans in a nursing home were found to be MRSA positive (nasal colonization) [2]. A decade ago, geriatric rehabilitation clinics have reported colonization rates ranging from 4.8% [3] to 9.8% [4]. It has also been shown that MRSA positive have a significantly longer length of stay (LOS) than MRSA negative rehabilitants [4,5]. Strict isolation of these patients is still recommended [6-8] but raises ethical concerns [9]. Isolation may cause psychological distress like depression and anxiety [10].

Risk factors for MRSA colonization are: former colonization with MRSA, hospitalization in the past, mechanical ventilation, antibiotic therapy, co-morbidity, tracheostomy, renal failure, and chronic skin infections [11-13]. It is evident that neurological early rehabilitation patients carry many of these risk factors, in particular hospitalization including long lasting intensive care therapy, mechanical ventilation, tracheostomy, antibiotic therapy (due to aspiration pneumonia), and co-morbidity [14,15]. However, only little data is available on MRSA incidence and prevalence in neurological early rehabilitation. In the BDH Clinic Hessisch Oldendorf, we have found 6.6% MRSA positive patients on intensive and intermediate care wards [16], and a recent multicenter study revealed a rate of 14.5% among ten large neurological early rehabilitation facilities in Germany [17].

It is hyothesized that strict isolation due to MRSA colonization limits rehabilitation, but there are no studies focusing on the outcome of MRSA colonized neurological rehabilitation patients, yet.

## Methods

The BDH Clinic Hessisch Oldendorf is a neurological hospital (including stroke unit and intensive care units) and rehabilitation facility in Northern Germany with more than 100 neurological early rehabilitation beds. With respect to MRSA the clinic is practicing a strict “search and destroy” strategy. All patients are systematically screened on admission (nose and orophharynx) using a polymerase chain reaction (PCR). If positive, a traditional culture is done to confirm MRSA colonization. All MRSA carriers are strictly isolated and nasal decolonization with mupirocin is initiated. Isolation will be continued until three subsequent samples are negative (first sample three days after last administration of mupirocin).

To find out whether MRSA colonization has any impact on outcome parameters, medical records of n = 569 neurological early rehabilitation patients admitted to the BDH Clinic Hessisch Oldendorf in 2010 have been reviewed. Barthel index (BI) [18], Early Rehabilitation Barthel Index (ERBI) [19], Glasgow Coma Scale (GCS) [20], Coma Remission Scale (CRS) [21], and Early Functional Abilities (EFA) [22] on admission have been included in the analysis. As major outcome parameters, BI and ERBI have been recorded. In addition, length of stay (LOS), morbidity (number of co-diagnoses and Patient Clinical Complexity Level – PCCL [14]), and duration of physiotherapy, ergotherapy, speech therapy, and cognitive therapy have been analyzed.

Statistical analyses included t-tests for independent samples, univariate analyses of variance, and bivariate Pearson correlations. Differences were regarded as significant with p < 0.05.

Local ethics committee (BDH-Clinic Hessisch Oldendorf) had no objections because the study was a retrospective database analysis, only (no intervention).

## Results

Among 569 neurological early rehabilitation patients (n = 304 men, n = 265 women), n=, n = 74 (13.0%) were tested MRSA positive (nasal and/or oropharynx colonization) on admission. All MRSA positive patients were strictly isolated for a mean of 31.5 (26.7) days. Most patients admitted to neurological early rehabilitation suffered from an ischemic stroke (Table 1).

**Table 1:** Main diagnoses of MRSA positive and negative neurological early rehabilitation patients

	**MRSA positive**	**MRSA negative**	**Sum**
**Ischemic stroke**	23	241	264
**Intracranial hemorrhage**	19	91	110
**Hypoxia**	6	13	19
**Polyneuropathy/Guillian-Barre-Syndrome**	4	14	18
**Brain injury**	2	14	16
**Brain tumour**	2	14	16
**Spinal injury**	1	6	7
**Other main diagnosis**	17	102	119
**Sum**	74	495	569

**Figure 1:**
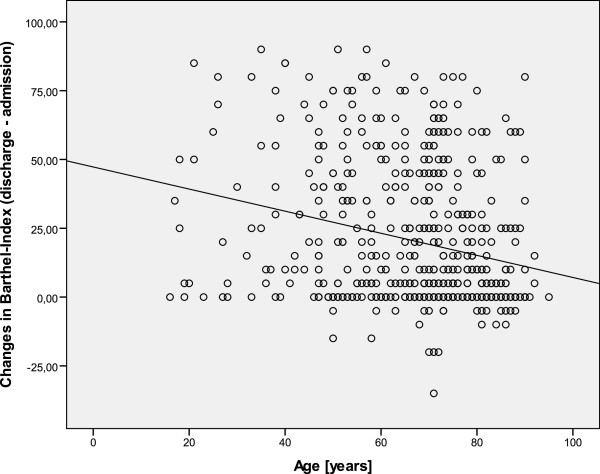
Age correlated negatively with changes in Barthel Index (discharge minus admission).

MRSA positives were not older than negative patients and length of stay (LOS) in referring (primary) hospitals was not different, either (Table 2). Age correlated negatively with changes in BI (discharge minus admission), r=-0.258, p<0.001, Figure 1. LOS in neurological rehabilitation was considerably longer among MRSA positives (Table 2). BI, ERI, GCS, and EFA on admission were worse in the MRSA group, and number of co-diagnoses and PCCL among these patients were significantly higher (Tables 2 and 3). Improvements in BI were significantly smaller in the MRSA positive group: +11.9 (20.3) vs. +21.8 (25.7), p < 0.01.

**Table 2:** Characteristics of MRSA positive and negative neurological early rehabilitation patients

	**MRSA positive**	**MRSA negative**	p-value*
**Age [years]**	65.8 (15.1)	67.0 (15.8)	n.s.
**Length of stay (LOS) – referring hospital [days]**	32.8 (42.9)	34.4 (260.5)	n.s.
**LOS – neurological early rehabilitation [days]**	63.7 (37.1)	25.8 (24.5)	< 0.001
**LOS – entire neurological rehabilitation [days]**	75.0 (42.5)	46.8 (47.1)	< 0.001
**Number of co-diagnoses [n]**	20.5 (5.1)	13.3 (5.5)	< 0.001
**Barthel Index (BI) on admission [0 to 100]**	13.6 (9.9)	25.6 (24.1)	< 0.001
**Barthel index on discharge [0 to 100]**	25.5 (21.2)	47.4 (31.0)	< 0.001
**Early Rehabilitation Index (ERI) on admission [−325 to 0]**	−80.1 (59.5)	−47.9 (47.6)	< 0.001
**ERI on discharge [−325 to 0]**	−47.3 (51.4)	−26.0 (35.4)	< 0.001
**Coma Remission Scale (CRS) [0 to 24]**	11.0 (6.2)	14.0 (6.8)	n.s.
**Glasgow Coma Scale (GCS) [3 to 15]**	9.5 (3.2)	12.0 (3.3)	< 0.001
**Early functional abilities (EFA) – vegetative [4 to 20]**	8.6 (3.0)	12.4 (7.0)	< 0.001
**EFA – faciooral [4 to 20]**	9.2 (5.0)	15.4 (5.3)	< 0.001
**EFA – sensorymotor [7 to 35]**	14.4 (6.9)	22.8 (8.2)	< 0.001
**EFA – cognitive [5 to 25]**	13.3 (6.4)	18.7 (5.2)	< 0.001
**Physiotherapy [min/day]**	42.5 (12.7)	36.6 (10.2)	< 0.001
**Ergotherapy [min/day]**	30.3 (1.9)	31.2 (4.7)	n.s.
**Speech therapy [min/day]**	24.1 (5.8)	27.2 (5.7)	< 0.001
**Cognitive therapy [min/day]**	34.9 (6.1)	42.9 (9.4)	< 0.001
**Total main therapies [min/day]**	131.6 (16.6)	140.2 (18.7)	< 0.001

*t-tests for independent samples, n.s. = not significant (p > 0.05).

**Table 3:** PCCL (Patient Clinical Complexity Level) of MRSA positive and negative patients

		**MRSA**		**Sum**
		**Negative**	**Positive**	
PCCL	0	35	0	35
	1	15	0	15
	2	43	3	46
	3	255	10	265
	4	147	61	208
Sum		495	74	569

χ^2^ = 78.1 (p < 0.001).

MRSA positive neurological early rehabilitation patients had significantly more physiotherapy and less speech and cognitive therapy per day (Table 2). Ergotherapy did not differ between the groups. Altogether, MRSA carriers had about 11 min less therapy per day than negative patients (Table 2). Because of significantly longer LOS of MRSA positive patients, the overall sum of therapeutic procedures during early rehabilitation was considerably higher in this group.

A univariate analysis of variance was performed using the following model: changes in BI (discharge minus admission) as dependent variable; colonization with MRSA and PCCL as categorical independent variables; age, BI on admission, GCS on admission, CRS on admission, duration of isolation, physiotherapy, ergotherapy, speech therapy, and cognitive therapy per day as independent covariates. This model explained 69.1% of the data variation (p < 0.001). PCCL had a highly significant influence (p < 0.001), but not MRSA colonization itself. Age (p < 0.05), physiotherapy per day (p < 0.01), and cognitive therapy per day (p < 0.05) also had a significant influence on changes in BI. CRS, GCS, ergotherapy, and speech therapy did not.

## Discussion

MRSA colonization is a growing problem in neurological rehabilitation. Recent studies suggest that the prevalence of MRSA carriers among early rehabilitation patients ranges from 6.6% to 14.5% [16,17]. Colonization with MRSA may limit neurological early rehabilitation because patients need to be isolated. MRSA carriers must not participate in group therapies which may lead to intensive human-resource allocation and less therapy for this cohort of rehabilitants. There are, however, no studies available focusing on the impact of MRSA colonization on the outcome of early rehabilitation patients, yet.

In the present study, there was a rate of 13.0% MRSA carriers on admission to the early rehabilitation facility which is in line with previously published results [17]. Patients had to be isolated for nearly half of their stay in neurological early rehabilitation (31.5 days isolation vs. 63.7 days LOS early rehabilitation). This finding suggests that decolonization procedures (local treatment with mupirocin [6]) are successful.

It is well known that age has an impact on the outcome of neurological patients, e.g. after stroke [23]. The present study also demonstrated a negative correlation between age and improvement in activities of daily living (BI) suggesting that age is a risk factor for poor neurological outcome. However, MRSA positive and negative groups did not differ with respect to age.

In line with previous studies, MRSA positive patients had a higher morbidity (PCCL, number of co-diagnoses), but LOS in the referring (primary) hospital was not different between both groups. Functional status (Barthel index, ERBI, CRS, EFA, GCS) was worse in the MRSA group on admission. LOS was significantly longer among MRSA carriers confirming previous findings [4,5].

Surprisingly, overall sum therapy was significantly larger in the MRSA group which can be explained by longer LOS in this cohort. Intensity of therapy (min per day), however, was slightly smaller among MRSA carriers (131.6 vs. 140.2 min/day). While there was even more physiotherapy per day in the MRSA colonized group, speech and cognitive therapy were done less frequently. Ergotherapy did not differ. This finding suggests that MRSA colonized patients do not necessarily receive less therapy than MRSA negative rehabilitants. Even so, the outcome of MRSA carriers was worse. How can this finding be explained? It is well known that low functional status on admission and co-morbidity are risk factors for poor outcome [14,24]. It emerges from literature that BI in the early phase is a strong predictor for long-term functional outcome [24]. Poor outcome among MRSA carriers may be explained by worse functional status on admission. In addition, lower BI and ERBI values on admission account for longer LOS among MRSA carriers [14,25]. This hypothesis is confirmed by a univariate analysis of variance: It turned out that BI improvement was strongly influenced by PCCL (as a measure of morbidity), age, and BI on admission. MRSA colonization itself had no independent influence on BI changes. Further studies on this topic are encouraged.

## Conclusions

Outcome of MRSA colonized neurological early rehabilitation patients is worse than functional independence of MRSA free patients. This finding cannot be explained by isolation effects, e.g. less therapy, but by significantly worse functional status and morbidity of MRSA carriers on admission.

## Competing interests

The author declares that he has no financial or non-financial competing interests.

## Pre-publication history

The pre-publication history for this paper can be accessed here:

http://www.biomedcentral.com/1471-2377/14/34/prepub

## References

[B1] Aeilts GD Sapico FL Canawath HN Malik GM Montgomerie JZ Methicillin-resistant Staphylococcus aureus colonization and infection in a rehabilitation facility J Clin Microbiol 1982 16 218 223 6922133 10.1128/jcm.16.2.218-223.1982 PMC272333

[B2] Cederna JE Terpenning MS Ensberg M Bardley SF Kauffman CA Staphylococcus aureus nasal colonization in a nursing home: eradication with mupirocin Infect Control Hosp Epidemiol 1990 11 13 16 10.2307/30144250 2105352

[B3] Heudorf U Bremer V Heuck D MRSA-Besiedelung bei Bewohnern von Alten- und Pflegeheimen sowie bei Patienten einer geriatrischen Rehabilitationsklinik in Frankfurt am Main 1999 Gesundheitswesen 2001 63 447 454 10.1055/s-2001-15924 11507671

[B4] Morrison L Stolarek I Does MRSA affect patient outcomes in the elderly? A retrospective pilot study J Hosp Infect 2000 45 169 171 10.1053/jhin.2000.0727 10860695

[B5] Hassouna H Haq EU Gall A MRSA colonisation in spinal cord injury: implications on patients rehabilitation Acta Orthop Belg 2008 74 528 530 18811038

[B6] Minary-Dohen P Bailly P Bertrand X Talon D Methicillin-resistant Staphylococcus aureus (MRSA) in rehabilitation and chronic-care-facilities: what is the best strategy? BMC Geriatr 2003 3 5 10.1186/1471-2318-3-5 14672540 PMC317303

[B7] Webber KL Macpherson S Meagher A Hutchinson S Lewis B The impact of strict isolation on MRSA positive patients: an action-based study undertaken in a rehabilitation center Rehabil Nurs 2012 37 43 50 10.1002/RNJ.00007 22271221

[B8] Kommission für Krankenhaushygiene und Infektionsprävention am RKI Bundesgesundheitsbl Gesundheitsforsch Gesundheitsschutz 1999 42 954 958 10.1007/s001030050227 28246721

[B9] Pike JH McLean D Ethical concerns in isolating patients with methicillin-resistant Staphylococcus aureus on the rehabilitation ward: a case report Arch Phys Med Rehabil 2002 83 1028 1030 10.1053/apmr.2002.33108 12098167

[B10] Tarzi S Kennedy P Stone S Evans M Methicillin-resistant Staphylococcus aureus: psychological impact of hospitalization and isolation in an older adult population J Hosp Infect 2001 49 250 254 10.1053/jhin.2001.1098 11740872

[B11] Vovko P Retelj M Cretnik TZ Jutersek B Harlander T Kolman J Gubina M Risk factors for colonization with MRSA in a long-term care facility in Slovenia Infect Control Hosp Epidemiol 2005 26 191 195 10.1086/502525 15756891

[B12] Aizen E Ljubuncic Z Ljubuncic P Aizen I Potasman I Risk factors for MRSA colonization in a geriatric rehabilitation hospital J Gerontol A Biol Sci Med Sci 2007 62 1152 1156 10.1093/gerona/62.10.1152 17921430

[B13] Torres K Sampathkumar P Predictors of methicillin-resistant Staphylococcus aureus colonization at hospital admission Am J Infect Control 2013 41 1043 1047 10.1016/j.ajic.2013.02.013 23706830

[B14] Rollnik JD Janosch U Current trends in the length of stay in neurological early rehabilitation Dtsch Arztebl Int 2010 107 286 292 20467554 10.3238/arztebl.2010.0286 PMC2868985

[B15] Oehmichen F Ketter G Mertl-Rötzer M Platz T Puschendorf W Rollnik JD Schaupp M Pohl M Weaning from prolonged mechanical ventilation in neurological weaning units: an evaluation of the German Working Group for early Neurorehabilitation Nervenarzt 2012 83 1300 1307 10.1007/s00115-012-3600-z 22814635

[B16] Rollnik JD Challenges for neurological rehabilitation in Germany Akt Neurol 2009 36 368 371 10.1055/s-0029-1220390

[B17] Thomas R MRSA in early rehabilitation – incidence, prevalence and morbidity Neurol Rehabil 2013 19 118 122

[B18] Mahoney FI Barthel DW Functional evaluation: the Barthel index Md State Med J 1965 14 61 65 14258950

[B19] Rollnik JD The Early Rehabilitation Barthel Index (ERBI) Rehabilitation (Stuttg) 2011 50 408 411 10.1055/s-0031-1273728 21626475

[B20] Teasdale G Jennett B Assessment of coma and impaired consciousness, a practical scale Lancet 1974 2 81 83 4136544 10.1016/s0140-6736(74)91639-0

[B21] Ortega-Suhrkamp E von Wild KR Standards of neurologic-neurosurgical early rehabilitation–a concept of the study group neurological-neurosurgical early rehabilitation Acta Neurochir Supp 2002 79 11 19 10.1007/978-3-7091-6105-0_2 11974974

[B22] Alvsåker K Walther SM Kleffelgård I Mongs M Drægebø RA Keller A Inter-rater reliability of the early functional abilities scale J Rehabil Med 2011 43 892 899 21879231 10.2340/16501977-0855

[B23] Kwakkel G Kollen BJ Predicting activities after stroke: what is clinically relevant? Int J Stroke 2013 8 25 32 10.1111/j.1747-4949.2012.00967.x 23280266

[B24] Pettersen R Dahl T Wyller TB Prediction of long-term functional outcome after stroke rehabilitation Clin Rehabil 2002 16 149 159 10.1191/0269215502cr482oa 11911513

[B25] Rollnik J Barthel-index as a length of stay predictor in neurological rehabilitation Rehabilitation (Stuttg) 2009 48 91 94 10.1055/s-0029-1202294 19421940

